# Antimalarial Activity and Safety Profile of Gaysorn‐Tang‐Ha (GSTH): A Thai Herbal Recipe Evaluated in an ICR Model

**DOI:** 10.1155/sci5/5638925

**Published:** 2026-05-07

**Authors:** Arisara Phuwajaroanpong, Chuchard Punsawad, Walaiporn Plirat, Atthaphon Konyanee, Parnpen Viriyavejakul, Abdi Wira Septama, Prapaporn Chaniad

**Affiliations:** ^1^ Center of Excellence in Tropical Pathobiology, Walailak University, Nakhon Si Thammarat, 80160, Thailand, wu.ac.th; ^2^ Department of Medical Technology, School of Allied Health Sciences, Walailak University, Nakhon Si Thammarat, 80161, Thailand, wu.ac.th; ^3^ Department of Medical Sciences, School of Medicine, Walailak University, Nakhon Si Thammarat, 80160, Thailand, wu.ac.th; ^4^ Department of Tropical Pathology, Faculty of Tropical Medicine, Mahidol University, Bangkok, 10400, Thailand, mahidol.ac.th; ^5^ Research Center for Pharmaceutical Ingredient and Traditional Medicine, National Research and Innovation Agency (BRIN), Cibinong Science Center, Bogor, 16915, Indonesia

## Abstract

Natural compounds have gained considerable attention in the search for novel therapeutic agents, particularly for infectious diseases such as malaria. Traditional herbal medicines offer promising alternatives to address the growing challenge of drug‐resistant *Plasmodium* strains. This study evaluated the antimalarial activity and safety profile of a Thai herbal recipe, Gaysorn‐Tang‐Ha (GSTH), or five‐flower remedy, using in vivo mouse models, including 4‐day suppressive tests and acute toxicity assessments. Furthermore, gas chromatography–tandem mass spectrometry (GC–MS/MS) was employed to characterize the chemical constituents of the ethanolic extract. Chemical profiling revealed that the GSTH extract predominantly contained terpenoid compounds and fatty acid derivatives, with squalene identified as a major constituent. In antimalarial assays, GSTH extract exhibited dose‐dependent suppression of parasitemia, with suppression rates of 18.31%, 47.31%, and 61.49% at doses of 200, 400, and 600 mg/kg body weight, respectively. Parasitemia suppression in GSTH‐treated mice at all doses was significantly higher (*p* < 0.05) than in the negative control. Acute toxicity testing revealed no observable physical or behavioral abnormalities in GSTH‐treated mice compared to the normal control group during 14 days. Additionally, there were no significant changes in body weight, liver, or kidney enzyme levels. Histopathological analysis of liver and kidney tissues showed no evidence of tissue damage, further supporting the safety of the extract. In conclusion, GSTH demonstrated significant dose‐dependent antimalarial activity, particularly at 600 mg/kg body weight, without observable signs of toxicity at doses up to 2 g/kg body weight. These findings indicate that the ethanolic GSTH extract exhibits moderate in vivo antimalarial activity with no evident acute toxicity under the conditions tested. The results provide preliminary evidence supporting further pharmacological and mechanistic investigations to better define its therapeutic potential.

## 1. Introduction

Herbal medicine has played a pivotal role in the treatment and prevention of various diseases, including malaria, since ancient times. It remains a valuable source of bioactive compounds with potential for modern drug development [1, 2]. Notably, plant‐derived alkaloids such as quinine and quinidine inhibit the malaria parasite’s ability to detoxify heme, a toxic byproduct of hemoglobin digestion in red blood cells [3]. Likewise, artemisinin, a sesquiterpene lactone from *Artemisia annua*, exerts its antimalarial effects through the generation of reactive oxygen species (ROS), leading to mitochondrial membrane depolarization, mitochondrial dysfunction, activation of caspase‐like enzymes, and DNA fragmentation, resulting in apoptotic cell death of the parasite [4, 5]. Over the past 3 decades, the acceptance of plant‐based treatments has significantly increased [6]. Traditional medicines remain the primary healthcare choice in many developing countries, particularly in remote and rural areas, owing to their cultural acceptance, accessibility, and affordability [1]. However, despite their widespread use, many traditional herbal medicines, including the Thai herbal recipe Gaysorn‐Tang‐Ha (GSTH), or five‐flower remedy, have received limited comprehensive scientific investigation to confirm their efficacy. In recent years, several Thai polyherbal formulations have been systematically evaluated for antimalarial activity. The Prabchompoothaweep remedy demonstrated moderate in vitro activity with low cytotoxicity and showed parasite suppression in murine models [7, 8]. Similarly, the Chan‐Ta‐Lee‐La and Pra‐Sa‐Chan‐Dang formulations exhibited potent in vitro activity and significant dose‐dependent reductions in parasitemia in standard in vivo assays, without evidence of acute toxicity. In addition, aqueous extracts of Trisamo and Jatu‐Phala‐Tiga have shown dose‐dependent in vivo antimalarial activity in *Plasmodium berghei*‐infected mice, with no observable acute toxicity at doses up to 2 g/kg, supporting their potential as antimalarial candidates [9]. These findings highlight the therapeutic potential of Thai polyherbal remedies and emphasize the importance of rigorous in vivo efficacy and safety evaluations for each specific formulation [10].

In Thai traditional medicine, GSTH is referred to as the “Five sacred pollens.” This recipe consists of five types of flowers: *Jasminum sambac* Ait (family Oleaceae), *Mimusops elengi* L. (family Sapotaceae), *Mesua ferrea* L. (family Calophyllaceae), *Nelumbo nucifera* (family Nelumbonaceae), and *Mammea siamensis* (family Calophyllaceae). This recipe is well‐known for its fragrant, cooling taste and has been traditionally used for a variety of therapeutic purposes, including treating dizziness, strengthening the heart, nourishing pregnancy, relieving internal heat, and promoting better sleep [11]. Additionally, GSTH exhibits antioxidant and anti‐inflammatory properties [11, 12]. More importantly, the ethanolic extract of GSTH has significant in vitro antimalarial activity, with an IC_50_ value of 2.8 ± 0.3 μg/mL, indicating highly potent activity against the *P. falciparum* K1 strain [13].

Despite these promising results, the current evidence is limited to in vitro assays. Although such assays are useful for preliminary screening, they cannot reliably predict in vivo efficacy because they do not account for host–parasite interactions, pharmacokinetics, metabolism, immune responses, or potential systemic toxicity [14]. To date, no study has evaluated the in vivo antimalarial activity or safety profile of GSTH in a validated animal model. Consequently, a significant translational gap remains between its demonstrated in vitro potency and potential therapeutic application. Therefore, in vivo investigation using an appropriate malaria model is essential to validate the efficacy and safety of GSTH.

Malaria remains a significant global health challenge, particularly in tropical and subtropical regions, where the emergence of drug‐resistant *Plasmodium* strains has undermined the efficacy of current antimalarial treatments [13, 15]. The treatment of five human *Plasmodium* spp., especially *P. falciparum* and *P. vivax*, depends on antimalarial drugs adapted to regional drug resistance situations [16]. Artemisinin‐based combination therapies (ACTs) are the cornerstone of treatment, especially for chloroquine‐resistant strains [16]. However, the increasing prevalence of artemisinin and partner drug resistance has raised significant concerns. Partial artemisinin resistance has been reported in the Greater Mekong subregion (GMS), particularly in Laos, Myanmar, Thailand, and Vietnam, as well as in several African countries, including Eritrea, Rwanda, Uganda, and the United Republic of Tanzania [15, 17]. This resistance has resulted in treatment failures associated with the ACTs, including artemether‐lumefantrine (AL), dihydroartemisinin‐piperaquine (DHA‐PPQ), artesunate‐amodiaquine (AS‐AQ), and artesunate‐pyronaridine (AS‐PY) in the WHO African region, as well as with AL, DHA‐PPQ, AS‐PY, artesunate‐mefloquine, and artesunate‐sulfadoxine‐pyrimethamine in the WHO South‐East Asia region [15, 18–20].

The rise in parasite resistance has contributed to more severe symptoms, resulting in higher mortality rates. Malaria often presents with symptoms similar to the common cold, including fever, chills, malaise, vomiting, coughing, dizziness, and headaches [21]. However, infection with the malaria parasite, particularly *P. falciparum*, can result in severe complications, such as severe anemia, neurological complications, hypoglycemia, respiratory distress, renal failure, liver damage, and cerebral malaria, particularly in children under 5 years of age and adolescents [21, 22]. According to the World Malaria Report 2024, approximately 263 million malaria cases were recorded worldwide, marking an increase of 11 million cases from the previous year. This resulted in approximately 619,000 fatalities in 2023, indicating an ongoing challenge in controlling the disease globally [15]. Furthermore, the emergence and spread of antimalarial drug resistance pose a significant threat to the effectiveness of current treatment strategies, particularly in malaria‐endemic regions of tropical and subtropical areas, including rural communities. Malaria disproportionately affects economically disadvantaged and remote areas, where limited access to healthcare presents significant challenges to treatment. In such regions, alternative treatments may offer practical and culturally acceptable solutions.

The development of new antimalarial agents, especially those that are accessible, affordable, and effective, is urgently needed to address the persistent burden of malaria and the growing threat of drug‐resistant *Plasmodium* strains. GSTH is a promising traditional herbal recipe that has demonstrated strong in vitro antimalarial activity, indicating its potential for further development. Given the absence of in vivo validation, systematic preclinical evaluation is necessary to determine whether the observed in vitro activity of GSTH translates into therapeutic efficacy in a living host. The standard 4‐day suppressive test in *P. berghei*‐infected mice provides a well‐established model for assessing blood‐stage antimalarial activity. This study aimed to evaluate the in vivo antimalarial efficacy of GSTH by assessing its suppressive activity, safety profile, and phytochemical characteristics. By extending previous in vitro findings to an in vivo murine malaria model, this study provides preclinical evidence supporting the further development of GSTH as a potential antimalarial candidate.

## 2. Materials and Methods

### 2.1. Plant Sourcing, Authentication, and Herbal Recipe Extraction

Five floral ingredients, including *J. sambac* Ait., *M. elengi* L., *M. ferrea* L., *N. nucifera*, and *M. siamensis,* were obtained from a licensed Thai medicinal drug store. Procurement details, including the date of purchase and lot numbers, were recorded to ensure traceability. The plant materials were identified based on diagnostic morphological characteristics and verified by an expert botanist through comparison with authenticated herbarium specimens. Subsequently, the five plant materials were then deposited in the Department of Medical Sciences, School of Medicine, Walailak University, Thailand, with voucher specimens of *J. sambac* (SMD187007002), *M. elengi* (SMD249006002), *M. ferrea* (SMD122007001), *N. nucifera* (SMD181001001), and *M. siamensis* (SMD122006002). The authorization for plant materials adhered to the Plant Varieties Protection, Department of Agriculture, Ministry of Agriculture and Cooperatives, Thailand. Each plant material was thoroughly washed with tap water and then dried in an oven at 60°C for 3 days. This temperature was selected to ensure efficient moisture removal while minimizing degradation of thermolabile bioactive compounds. After drying, the plant materials were finely ground using an electric herb grinder (Jincheng, model; SF, China). The GSTH remedy was prepared by mixing five herbal ingredients in equal portions of 20 g each, with a total weight of 100 g, to obtain a sufficient amount of crude extract. The mixture was extracted using the maceration method by soaking it in ethanol at a 1:10 (w/v) ratio. Ethanol was selected as the extraction solvent based on preliminary in vitro screening, which demonstrated that the ethanolic extract possessed superior antimalarial activity compared with the aqueous extract.

The extraction process was conducted for 72 h at 25°C, with occasional stirring. The extraction was then filtered through Whatman No. 1 filter paper to separate the liquid extract and residue. The residue underwent two additional extraction cycles with a 1:10 ratio of residue to fresh ethanol. After filtration, the combined filtrates were concentrated by a rotary evaporator (N‐1200B, EYELA) to obtain an ethanolic extract. The weight of the crude extract was measured to calculate the extraction yield before being stored in an airtight container in a refrigerator until further analysis and use in *in vivo* studies. The percentage yield was calculated using the following formula:

(1) percentage yield=weight of crude extractinitial weight of herbal recipe×100.

### 2.2. Phytochemical Analysis Using a Triple Quadrupole GC–MS/MS

Phytochemical identification was performed using a Triple Quadrupole GC–MS/MS (GC‐QQQ) equipped with a 7607A static headspace sampler (Agilent Technologies, Santa Clara). The instrument specifications included a 7890B GC system and a 7000C Mass Selective Detector (MSD) GC/MS Triple Quad with an electron ionization (EI) ion source. The GC column used was an HP‐5 ms (30 m × 0.25 mm, 0.25 μm). The injection volume of the ethanol‐diluted sample was 1 μL, and the inlet temperature was set to 250°C. The EI system was operated at an EI voltage of 70 eV, with the EI source temperature set to 250°C. The mass scanning range was 33–600 amu for the MSD. Helium was used as the carrier gas at a constant flow rate of 1 mL/min. The oven temperature program was as follows: initially set to 60°C and held for 2 min, then ramped to 150°C (Ramp 1) at a rate of 10°C/min, followed by a ramp to 300°C (Ramp 2) at a rate of 5°C/min. The final temperature of 300°C was maintained for a total of 55 min. Data acquisition was performed using MassHunter Workstation GC/MS Data Acquisition Version 10.1.49, and data processing was carried out using MassHunter Workstation Qualitative Analysis Version 10.0. The compounds were identified using the spectral database of the National Institute of Standards and Technology (NIST 2020), with identification based on peaks exhibiting a minimum of 80% similarity to entries in the database.

### 2.3. Mouse Experiments

Male ICR mice (20–30 g, 6–8 weeks old) were supplied by Nomura Siam International (Bangkok, Thailand). Only males were used to minimize potential variability associated with hormonal fluctuations in females, which may influence metabolic and behavioral outcomes. The mice underwent a one‐week acclimatization period under controlled laboratory conditions, including a stable room temperature of 23°C ± 2°C, humidity levels between 50% and 70%, and a 12‐h light/dark cycle. Throughout the experiments, the mice were provided food pellets and clean drinking water *ad libitum*.

This study adheres to the ethical principles and guidelines established for the use of animals in scientific research in Thailand, ensuring that all procedures comply with national regulations and uphold the welfare of the animals involved [23]. This study was approved by the Institutional Review Board (or Ethics Committee) of Walailak University, under the National Research Council of Thailand (NRCT), with protocol number WU‐ACUC‐67006. All handling and experimental procedures were carried out in strict accordance with ethical procedures.

### 2.4. Peters’ 4‐Day Suppressive Test

The antimalarial activity was evaluated using Peters’ 4‐Day Suppressive Test [24]. The *P. berghei* ANKA strain, a rodent malaria parasite, was obtained from BEI Resources, NIAID, NIH, and was originally provided by Thomas F. McCutchan. Thirty male ICR mice were randomly divided into six groups, with five mice in each. Group 1 served as the negative control. Groups 2 and 3 served as positive controls and were treated with artesunate (6 mg/kg body weight) and chloroquine (25 mg/kg body weight), respectively. Groups 4, 5, and 6 received the ethanolic extract of GSTH at doses of 200, 400, and 600 mg/kg body weight, respectively.

Donor mice were inoculated with *P. berghei* ANKA via intraperitoneal injection, and parasitemia levels were monitored daily using Giemsa‐stained thin smear microscopy. Once parasitemia reached 20%–30%, blood was collected, suspended in normal saline, and used to initiate the infection in mice for the suppressive test. Parasitemia was quantified by counting erythrocytes under oil immersion (100 ×), and total erythrocyte counts were determined using a hemocytometer. The concentration of parasitized erythrocytes was calculated as total erythrocytes/mL × (% parasitemia/100), and the suspension was adjusted with sterile normal saline to 5 × 10^7^ parasitized erythrocytes/mL, such that each mouse received 0.2 mL containing 1 × 10^7^ parasitized erythrocytes. A total of 30 mice were intraperitoneally injected with 0.2 mL of the diluted *P. berghei*‐infected blood. At 3, 24, 48, and 72 h post‐infection, the mice were orally administered test substances. Mice in the negative control group received the vehicle solution consisting of 7% Tween 20 and 3% ethanol in distilled water. Mice in the positive control groups received artesunate (6 mg/kg body weight) or chloroquine (25 mg/kg body weight). Mice in the three treatment groups received the ethanolic GSTH extract at doses of 200, 400, and 600 mg/kg body weight, respectively. All treatments were administered orally, and the administered volume was calculated individually according to the body weight of each mouse to achieve the intended mg/kg dose (Table 1). On Day 5 (96 h post‐infection), blood samples were collected via tail clip to prepare thin films that were then stained with Giemsa dye. Parasitemia levels were assessed by examination of Giemsa‐stained thin smears under a light microscope (Olympus CX31, Model CX31RBSFA, Tokyo, Japan) using oil immersion at 100 × magnification. The mice were anesthetized with 2% isoflurane. After confirming deep anesthesia by the absence of a withdrawal reflex upon foot stimulation, the animals were euthanized. The percentages of parasitemia and parasitemia suppression were calculated using the following equations:

(2) %parasitemia=number of infected red blood cellsnumber of total red blood cells×100,

(3) %suppression=mean parasitemia in negative group−mean parasitemia in experimental groupmean parasitemia in negative group×100.

**TABLE 1:** Grouping of mice in the 4‐day suppressive test.

Group number (*n* = 5/group)	Treatment (dose)
1. Negative control	7% Tween 20 and 3% ethanol in distilled water
2. Positive control‐I	Artesunate (6 mg/kg body weight)
3. Positive control‐II	Chloroquine (25 mg/kg bodyweight)
4. Testing group (low dose)	GSTH (200 mg/kg body weight)
5. Testing group (medium dose)	GSTH (400 mg/kg body weight)
6. Testing group (high dose)	GSTH (600 mg/kg body weight)

### 2.5. Acute Toxicity Test

The testing procedure followed the Organization for Economic Co‐operation and Development (OECD) guideline No. 425 for the testing of chemicals with a limit test at a dose of 2 g/kg body weight [25], with minor modifications. A normal control and a vehicle control group were included to distinguish treatment‐related effects from potential solvent‐related effects and to strengthen the interpretation of safety outcomes. Fifteen male ICR mice were randomly assigned to three groups: normal, negative, and testing. The normal group received a single oral dose of 0.2 mL of normal saline solution. The negative group received an oral dose of 0.2 mL of vehicle solution (7% Tween 20 and 3% ethanol in distilled water). The testing group received a single oral dose of 0.2 mL of 2 g/kg body weight of GSTH extract. Before administration, the mice were weighed and deprived of both food and water for 3 h, followed by oral administration of the respective solutions. Abnormalities were observed immediately after oral administration and subsequently monitored twice daily for 14 consecutive days. The observed behavioral and physiological abnormalities included neurological, physiological, and general health indicators, such as alterations in activity levels (ranging from hypoactivity to hyperactivity), tremors, convulsions, postural abnormalities, grooming behavior, diarrhea, changes in skin, fur, and eye conditions, and mortality. Mice were weighed and anesthetized with 2% isoflurane. After deep anesthesia was confirmed by the absence of a withdrawal reflex upon foot stimulation, blood samples were collected via cardiac puncture, and the animals were subsequently euthanized. Liver and kidneys were carefully harvested for histological examination with hematoxylin and eosin (H&E) staining. The blood samples were analyzed to assess liver and kidney function, including aspartate aminotransferase (AST), alanine aminotransferase (ALT), alkaline phosphatase (ALP), blood urea nitrogen (BUN), and creatinine using a Beckman Coulter AU480 automated chemistry analyzer (Beckman Coulter, USA).

### 2.6. Histopathological Analysis of the Liver and Kidneys in Acute Toxicity Tests

Histopathological examinations were performed as previously described [26]. Briefly, the liver and kidneys obtained from mice in acute toxicity tests were immediately fixed in 10% (v/v) formalin following harvesting. During dehydration, the tissues were gradually immersed in increasing concentrations of ethanol (70%, 90%, and 100%) to remove the water content. The tissues were then immersed in xylene and embedded in molten paraffin wax within molds. The paraffin‐embedded tissue blocks were sectioned at a thickness of 5 μm using a microtome, and the sections were subsequently placed onto glass slides. The tissue sections were immersed in xylene to remove the paraffin wax and were then rehydrated by passing through a series of decreasing ethanol concentrations (100%, 90%, and 70%) before being rinsed in distilled water. The tissue sections were stained with H&E solutions, dehydrated through increasing ethanol concentrations, and cleared with xylene. The microscopic examination was performed under a light microscope at 40× magnification.

### 2.7. Statistical Analysis

Statistical analyses were conducted using SPSS for Microsoft Windows (Version 17.0; IBM, Armonk, NY, USA). Data are presented as the mean ± standard error of the mean (mean ± SEM). Normality of distribution was assessed, and subsequent analysis was performed using one‐way analysis of variance (ANOVA) followed by Tukey’s post hoc multiple comparison test. A *p* < 0.05 was considered statistically significant.

## 3. Results

### 3.1. Characterization and Percentage Yield of Ethanolic GSTH Extracts

The ethanolic GSTH extract appeared as a dark brown, sticky, and thick paste. The yield of the ethanolic extracts of GSTH was 15.62% relative to the initial weight of the dried plant materials.

### 3.2. Identification and Composition of Ethanolic GSTH Extracts by GC–MS/MS Analysis

**FIGURE 1:**
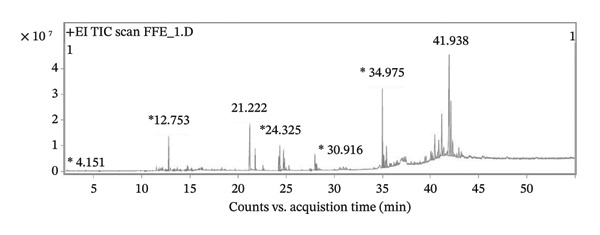
Chromatographic profile of the GSTH recipe.

Analysis of the ethanolic GSTH extract identified 35 compounds (Table 2), with the chromatographic profile presented in Figure 1. Upon examining the overall composition, the extract was found to contain a significant proportion of terpenoids (51.43%) and fatty acid derivatives (28.57%). Notably, 18 compounds were terpenoids, representing the predominant chemical group, whereas 10 compounds were fatty acid derivatives. Additionally, two phenolic compounds were detected in the extract: 3,5‐di‐tert‐butylphenol and 2‐hydroxy‐1,8‐naphthyridine, with retention times (RTs) of 13.369 and 22.577 min, respectively. The extract also contained styrene, classified as an aromatic hydrocarbon, along with alkanes, including 2‐hydroxy‐1,8‐naphthyridine and octadecane.

**TABLE 2:** Identification of compounds in GSTH extracts using GC–MS/MS analysis.

No.	RT (min)	Compounds	Formula	Molecular weight	Peak area (%)
1	3.303	3‐Methylbutanoic acid	C_5_H_10_O_2_	102	0.14
2	3.438	2‐Methylbutanoic acid	C_5_H_10_O_2_	102	0.22
3	4.151	Styrene	C_8_H_8_	104	0.49
4	7.619	Phenylethyl alcohol	C_8_H_10_O	122	0.41
5	11.490	alpha‐Copaene	C_15_H_24_	204	1.22
6	12.140	beta‐Caryophyllene	C_15_H_24_	204	1.19
7	12.424	Aromandendrene	C_15_H_24_	204	0.77
8	12.552	*cis*‐beta‐Farnesene	C_15_H_24_	204	0.31
9	12.640	Humulene	C_15_H_24_	204	0.44
10	12.753	Alloaromadendrene	C_15_H_24_	204	14.49
11	12.945	gamma‐Muurolene	C_15_H_24_	204	0.78
12	13.369	3,5‐Di‐tert‐butylphenol	C_14_H_22_O	206	1.28
13	13.540	gamma‐Cadinene	C_15_H_24_	204	0.84
14	13.659	delta‐Cadinene	C_15_H_24_	204	1.28
15	14.580	Spathulenol	C_15_H_24_O	220	0.67
16	14.696	Caryophyllene oxide	C_15_H_24_O	220	3.05
17	14.779	Hexadecane	C_16_H_34_	226	1.90
18	15.026	Isoaromadendrene epoxide	C_15_H_24_O	220	0.56
19	15.127	Humulene epoxide II	C_15_H_24_O	220	1.88
20	15.560	10,10‐Dimethyl‐2,6‐dimethylenebicyclo[7.2.0]undecan‐5beta‐ol	C_15_H_24_O	220	0.32
21	15.901	14‐Hydroxycaryophyllene	C_15_H_24_O	220	0.84
22	16.271	Mustakone	C_15_H_22_O	218	1.25
23	18.275	Octadecane	C_18_H_38_	254	1.27
24	19.703	Clovanediol	C_15_H_26_O_2_	238	1.08
25	21.222	Palmitic acid	C_16_H_32_O_2_	256	44.67
26	21.777	Ethyl hexadecanoate	C_18_H_36_O_2_	284	11.70
27	22.577	2‐Hydroxy‐1,8‐naphthyridine	C_8_H_6_N_2_O	146	2.84
28	22.693	alpha‐Kaurene	C_20_H_32_	272	0.94
29	24.212	Linoleic acid	C_18_H_32_O_2_	280	12.50
30	24.325	Linolenic acid	C_18_H_30_O_2_	278	24.13
31	24.822	Linolenic acid, ethyl ester	C_20_H_34_O_2_	306	7.46
32	24.981	Hexadecanamide	C_16_H_33_NO	255	2.06
33	25.268	Ethyl octadecanoate	C_20_H_40_O_2_	312	2.62
34	27.977	Oleamide	C_18_H_35_NO	281	10.98
35	34.975	Squalene	C_30_H_50_	410	53.59

The most abundant compound detected was squalene (RT; 34.975), belonging to the triterpenes class, followed by palmitic acid (a fatty acid, RT; 21.222), linolenic acid (a fatty acid, RT; 24.325), alloaromadendrene (a terpenoid, RT; 12.753), linoleic acid (a fatty acid, RT; 24.212), and ethyl hexadecanoate (a fatty acid ester, RT; 21.777).

### 3.3. Parasitemia Reduction and Suppression in the 4‐Day Suppressive Test

The parasitemia percentage of mice treated with the ethanolic GSTH extract is presented in Figure 2(a). The parasitemia percentage of the negative control was 24.76 ± 0.90. Mice treated with artesunate exhibited a parasitemia percentage of 1.18 ± 0.18, and those receiving the extract at 200 mg/kg, 400 mg/kg, and 600 mg/kg body weight showed parasitemia percentages of 20.22 ± 0.16, 13.05 ± 0.18, and 9.53 ± 0.17, respectively. Additionally, chloroquine at 25 mg/kg body weight resulted in the complete eradication of parasites. The parasitemia percentage in the negative control group was significantly higher than that in the groups of mice treated with the standard drugs and all doses of the extract (*p* < 0.05). However, no significant difference in the percentage of parasitemia was observed between the groups treated with chloroquine or artesunate (*p* > 0.05).

FIGURE 2Parasitemia (a) and suppression (b) percentages. Data are presented as mean ± SEM (*n* = 5 per group). Statistical comparisons (*p* < 0.05) were as follows: ^A^compared with the negative control group, ^B^compared with the positive control group receiving CQ, ^C^compared with the positive control group receiving artesunate, ^D^compared with GSTH 200 mg/kg body weight, ^E^compared with GSTH 400 mg/kg body weight, and ^F^compared with GSTH 600 mg/kg body weight.

**Figure:**
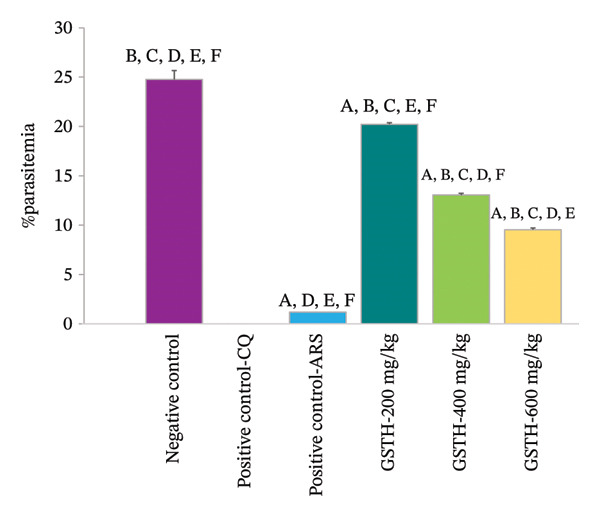
(a)

**Figure:**
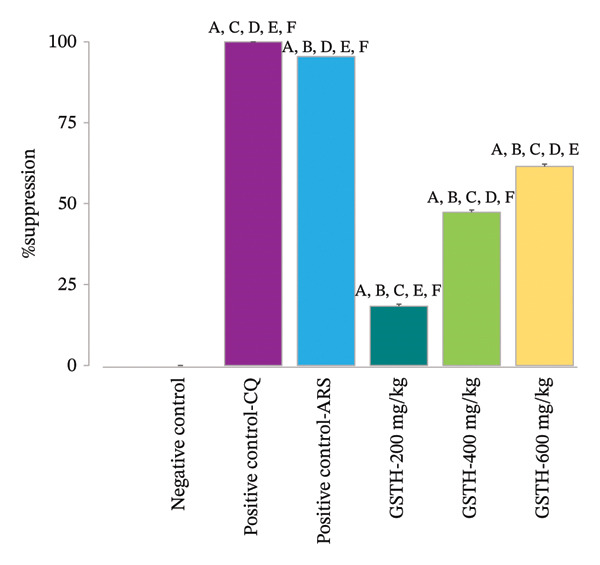
(b)

The percentage suppression of parasitemia is presented in Figure 2(b). Among all treatment groups, chloroquine exhibited the highest suppression (100%), demonstrating a significantly greater suppression compared to all other groups (*p* < 0.05), including artesunate. Artesunate produced 95.42% suppression, which was also significantly greater than that of the remaining treatment groups (*p* < 0.05). Furthermore, the GSTH extract demonstrated a significant, dose‐dependent suppression of *P. berghei* ANKA when compared to the negative control group (*p* < 0.05). The percentage suppressions were 18.31%, 47.31%, and 61.49% at 200, 400, and 600 mg/kg body weight, respectively. Notably, the suppression rates among different extract doses were also significantly different (*p* < 0.05).

### 3.4. Effects of Ethanolic GSTH Extract on the Physical and Behavioral Responses and Body Weight Changes in Acute Toxicity Assays

No behavioral or physiological abnormalities were observed in mice that received the vehicle or extract during 14 days compared to normal mice. Mice administered a single oral dose of 2 g/kg body weight of the extract exhibited normal behaviors, including proper posture, characterized by a balanced alignment of the head, body, and limbs. Mice also demonstrated normal active movement, with the ability to walk without ataxia. Grooming activity, including licking, biting, and scratching of their fur, was observed in all groups, indicating normal self‐care behaviors. Furthermore, all mice showed normal social interactions, engaging in exploratory behavior, mutual sniffing, grooming, and interaction within the group. Additionally, all mice exhibited normal physical health. No visible injury or signs of irritation were observed on the skin, and the eyes appeared clear, free from discharge or swelling. Moreover, normal feces were observed in all groups, with no signs of diarrhea. Throughout the experiment, no mortalities occurred, indicating that the LD_50_ of ethanolic GSTH was greater than 2 g/kg body weight.

No significant differences in body weight (*p* > 0.05) were observed among the normal, vehicle (7% Tween 20 and 3% ethanol in distilled water), and the extract‐treated groups from Day 1 to Day 7, Day 7 to Day 14, and Day 1 to Day 14 (Table 3).

**TABLE 3:** Body weight and percentage changes in acute toxicity tests.

Group	Body weight (g)			%Change		
	Day 1	Day 7	Day 14	Days 1–7	Days 7–14	Days 1–14
Normal	28.04 ± 0.78	35.91 ± 1.22	39.38 ± 1.53	28.03 ± 2.20	9.64 ± 1.40	40.39 ± 3.09
Vehicle	27.95 ± 0.53	35.00 ± 0.45	38.16 ± 0.48	25.44 ± 3.20	9.05 ± 0.89	36.72 ± 2.89
GSTH	31.80 ± 0.93	36.39 ± 0.60	42.42 ± 0.51	20.45 ± 1.96	10.99 ± 1.26	33.73 ± 3.12

*Note:* The data are presented as mean ± SEM (*n* = 5 per group). No significant differences (*p* < 0.05) were observed.

### 3.5. Effects of the Ethanolic GSTH Extracts on the Liver and Kidney Function Tests

The liver and kidney function parameters, including BUN, creatinine, AST, ALT, and ALP, assessed in the acute toxicity tests, are presented in Table 4. Kidney function markers (BUN and creatinine) showed no significant differences (*p* > 0.05) among the normal, vehicle (7% Tween 20 and 3% ethanol in distilled water), and GSTH‐treated groups. Similarly, liver function markers, including ALT and ALP, did not differ significantly (*p* > 0.05) among the normal, vehicle, and GSTH‐treated groups. However, AST levels were significantly lower (*p* < 0.05) in mice treated with the vehicle compared to both the normal and GSTH‐treated groups.

**TABLE 4:** Effects on the liver and kidney functions in acute toxicity tests.

Group	Liver function			Kidney function	
	AST (U/L)	ALT (U/L)	ALP (U/L)	BUN (mg/dL)	CREA (mg/dL)
Normal	124.80 ± 4.80^b^	44.20 ± 2.75	113.40 ± 5.22	12.60 ± 0.24	0.50 ± 0.99
Vehicle	110.80 ± 3.65^a,c^	44.40 ± 3.14	104.80 ± 6.84	10.00 ± 0.95	0.40 ± 0.19
GSTH	115.40 ± 4.99^b^	45.00 ± 4.00	107.60 ± 7.30	13.00 ± 0.00	0.42 ± 0.65

*Note:* Data are presented as mean ± SEM (*n* = 5 per group). Statistical comparisons were made as follows at *p* < 0.05.

^a^Compared with the normal group.

^b^The vehicle control group (7% Tween 20 and 3% ethanol in distilled water).

^c^The ethanolic GSTH‐treated group.

### 3.6. Histopathological Analysis of the Liver and Kidneys in Mice Subjected to Acute Toxicity Tests

**FIGURE 3:**
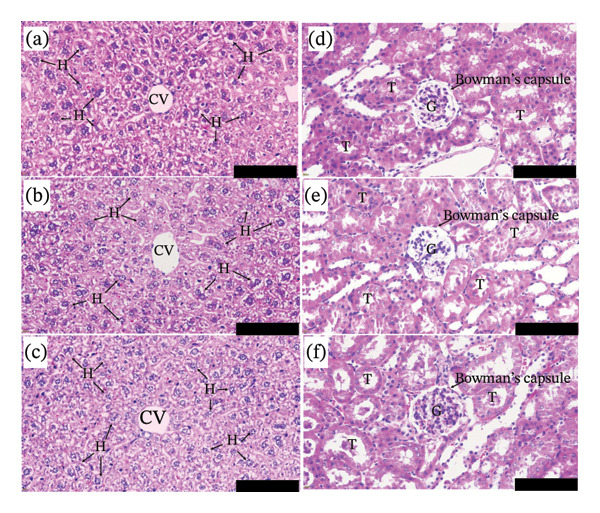
Histopathological evaluation of liver and kidney tissues using H&E staining. Images were captured at 40× magnification. Scale bar = 100 μm. CV, central vein; H, hepatocyte; T, renal tubule; G, glomerulus. Groups: (a) liver histology of control mice; (b) liver histology of the vehicle‐treated group, which received 7% Tween 20 and 3% ethanol in distilled water; (c) liver histology of mice treated with GSTH at 2 g/kg body weight; (d) kidney histology of control mice; (e) kidney histology of the vehicle‐treated group, which received 7% Tween 20 and 3% ethanol in distilled water; and (f) kidney histology of mice treated with ethanolic GSTH at 2 g/kg body weight.

The histopathological examination of the liver (Figures 3(a), 3(b), 3(c)) and kidneys (Figures 3(d), 3(e), 3(f)) in the acute toxicity tests is shown in Figure 3. In the liver, no signs of cell swelling, ballooning degeneration, or atrophy were observed in mice treated with the vehicle (Figure 3(b)) or GSTH (Figure 3(c)), indicating that hepatocyte morphology remained normal (Figure 3(a)). Additionally, no nuclear abnormalities, such as nuclear pleomorphism, multinucleation, or hyperchromatic changes, were detected in any group. Furthermore, no cytoplasmic hyaline inclusions, congestion, or necrosis were observed in any of the groups (Figures 3(a), 3(b), 3(c)).

Similarly, the histopathological assessment of the kidneys revealed no evidence of glomerular injury, such as basement membrane thickening, focal segmental glomerulosclerosis, or mesangial expansion, in any group (Figures 3(d), 3(e), 3(f)). No signs of tubular injury, including swollen tubular epithelial cells, debris accumulation in tubular lumens, or tubular vacuolization, were observed. Additionally, no congestion or inflammatory cell infiltration was detected in any group. These findings indicate that the ethanolic GSTH extract did not induce histopathological alterations in the liver or kidneys, supporting its safety profile.

## 4. Discussion

Malaria remains one of the most important tropical infectious diseases, posing a significant threat to global public health, particularly in endemic regions. The emergence and spread of drug‐resistant *Plasmodium* strains have further complicated malaria control efforts and underscored the urgent need for novel antimalarial agents. In response to this challenge, this study aimed to evaluate the antimalarial potential of a traditional Thai herbal recipe, GSTH, which has been used in folk medicine but has not yet been scientifically validated for in vivo antimalarial activity. Our results indicate that the ethanolic extract of GSTH possesses antimalarial activity in a dose‐dependent manner. Significant reductions in parasitemia were observed at all tested doses, with the highest suppression (61.49%) exhibited at 600 mg/kg body weight. Based on commonly accepted in vivo screening criteria, plant extracts producing at least 50% parasitemia suppression at 500 mg/kg are classified as having moderate antimalarial activity. Accordingly, the 61.49% suppression observed at 600 mg/kg indicates that GSTH demonstrates moderate in vivo antimalarial activity. Although the suppression was not as complete as standard antimalarial drugs such as chloroquine or artesunate, these findings provide preliminary in vivo evidence supporting the biological activity of GSTH rather than definitive therapeutic efficacy.

The observed reduction in parasitemia following treatment may be associated with the presence of the immunomodulatory properties of squalene, a principal component identified in the GSTH extract. However, this interpretation is based on GC–MS profiling, which provides semiquantitative data, and does not establish a direct causal relationship between squalene and the observed antimalarial activity. Squalene is a natural triterpene and a biosynthetic precursor to sterols such as cholesterol, with antioxidant, antitumor, antibacterial, and detoxification effects [27]. Furthermore, squalene potentiates immune responses by modulating both innate and adaptive immune mechanisms [27]. Its mechanisms of action include the activation of dendritic cells (DCs), modulation of T and B lymphocytes, stimulation of monocytes, and regulation of cytokine production, particularly tumor necrosis factor‐α (TNF‐α) and interleukin‐10 (IL‐10) [28–30]. During malaria infection, *Plasmodium* parasites employ diverse immune evasion strategies that compromise host immunity [31]. These strategies involve the suppression of DC function, resulting in diminished T cell proliferation, impaired CD8^+^ T cell activation, and triggered atypical activation patterns of DCs. Additionally, *Plasmodium* infection downregulates proinflammatory cytokine expression, thereby weakening the host’s ability to mount an effective immune response [31, 32].

Consequently, the immunostimulatory properties of squalene could potentially contribute to counteracting these immune evasion mechanisms. Nevertheless, this proposed contribution remains hypothetical, as the present study did not directly evaluate immunological or mechanistic pathways. In addition, in silico studies have identified squalene as a potential inhibitor of *P*. *falciparum* dihydroorotate dehydrogenase, a key enzyme involved in the parasite’s *de novo* pyrimidine biosynthesis pathway. This suggests that the antimalarial activity of the GSTH extract could potentially involve direct interference with parasite metabolism [33]. Malaria infection also induces oxidative stress, resulting in an imbalance between ROS and reactive nitrogen species (RNS) [34]. This oxidative stress arises primarily from hemoglobin degradation by the parasite and the host’s immune response. The elevated levels of ROS and RNS contribute to systemic and tissue oxidative damage, which is associated with severe complications such as cerebral malaria [35]. Squalene and linolenic acid, the major compounds identified in the GSTH extract, exhibit antioxidant properties. They enhance antioxidant defense systems by upregulating the activity of key antioxidant enzymes, including superoxide dismutase (SOD), catalase (CAT), and glutathione peroxidase (GPx), as well as by contributing to the overall antioxidant capacity [36–38]. Thus, ethanolic GSTH extract may have antioxidant properties, similar to other antimalarial agents, which may help mitigate oxidative damage and improve clinical outcomes.

Furthermore, in this study, the ethanolic extract of GSTH significantly suppressed parasitemia in a dose‐dependent manner, with the highest suppression observed at 600 mg/kg body weight. This finding suggests that the antimalarial efficacy of the extract may result from the synergistic interactions among its components, a phenomenon commonly observed in other polyherbal formulations [10]. Terpenoids, coumarins, and xanthones, compounds isolated from the flowers of *M. siamensis*, have antimalarial activity and are potential lead structures for the development of potent inhibitors targeting *P. falciparum* lactate dehydrogenase (*Pf*LDH) [39]. *M. ferrea* exhibits antimalarial activity, particularly in extracts derived from different plant parts. Leaf and bark extracts show significant, dose‐dependent suppression of *P*. *berghei* ANKA, with parasitemia reductions of 54.48% and 62.61%, respectively [40]. Additionally, compounds isolated from the leaves of *N. nucifera* show in vitro antimalarial activity against *P. falciparum* D6 and W2 clones [41]. Therefore, the antimalarial activity of the GSTH extract is likely multifactorial and may arise from the combined or synergistic effects of multiple phytochemicals identified in the extract. While squalene was one of the major constituents detected, its specific contribution to the overall activity cannot be determined based solely on GC–MS peak area percentages. Antimalarial activity was evaluated using the 4‐day suppressive (Peter’s) test, which primarily reflects early blood‐stage activity. As the curative model was not included, the findings represent a preliminary efficacy assessment. In addition, the specific molecular mechanisms underlying the observed activity were not investigated. Future studies incorporating curative models and mechanistic analyses are warranted to further define the therapeutic potential of the GSTH extract.

Acute oral toxicity studies are essential for the preliminary safety evaluation of new therapeutic candidates, as the liver and kidneys are the primary organs responsible for xenobiotic metabolism and excretion following oral administration. Therefore, the present study employed acute toxicity tests to assess the safety of the extract, focusing on the liver and kidneys. No signs of physical or behavioral changes were observed in any of the treated groups, indicating that the ethanolic GSTH extract did not induce observable toxicity under the tested conditions. Changes in body weight are key indicators of systemic toxicity, as significant weight loss may reflect adverse physiological or metabolic effects induced by the tested substance [42]. In the present study, no significant differences (*p* > 0.05) in body weight were observed among the normal control, vehicle control, and extract‐treated groups. These findings support the safety profile of the extract, indicating that it did not adversely affect feeding behavior or produce systemic toxicity.

In addition, histopathology and liver and kidney functions, including AST, ALT, ALP, BUN, and creatinine, were assessed, as these organs serve as the primary sites for extract metabolism and excretion. No significant differences (*p* > 0.05) were observed in the levels of AST, ALT, ALP, BUN, and creatinine compared to the normal control group, and no histopathological alterations were observed in any of the groups, suggesting that the GSTH extract does not induce hepatotoxicity or nephrotoxicity. Nevertheless, although there were no significant changes in liver and kidney functions overall, the vehicle control group exhibited significantly lower AST levels (*p* < 0.05) compared to both the normal control and GSTH‐treated groups. However, AST levels in all groups remained within the normal range [43]. The absence of mortality at 2 g/kg suggests that the LD_50_ is greater than this dose [25]. However, these findings are limited to single‐dose acute exposure and indicate only the absence of observable acute toxicity under the conditions of this study. Subacute, chronic, and repeated‐dose toxicity studies were not performed and are required to more comprehensively characterize the safety profile of the extract.

The low toxicological profile of the extract may be attributed to the presence of naturally occurring phytochemicals with well‐established safety margins. Several major constituents identified in the extract, such as squalene, exhibit minimal toxicity. Squalene is considered nontoxic owing to its lipid‐based chemical structure, despite being prone to oxidation [44]. Moreover, squalene is well tolerated in biological systems, and its structural resemblance to endogenous lipids facilitates efficient metabolism and clearance without inducing significant toxicity. It is also widely utilized in pharmaceutical formulations, including dietary supplements [37, 45]. Oral or parenteral administration of squalene in animal models and humans did not produce adverse effects, even at high doses [37, 45]. These findings collectively support the safety profile of the GSTH extract, which contained squalene as a main component. This suggests that the overall safety of the extract may result from the predominance of bioactive compounds with low toxicity. The second major compound identified in the ethanolic extract was palmitic acid, a saturated fatty acid that is widely present in various foods and traditional Chinese medicines. However, at high concentrations, palmitic acid is associated with lipotoxic effects, such as hepatocellular steatosis [46]. In addition, lipotoxic effects can lead to cellular dysfunction and death across multiple cell types. Moreover, palmitic acid reduces the viability of human proximal tubular epithelial cells [47].

Nonetheless, in the present study, despite the presence of palmitic acid as the second most abundant component in the extract, no significant alterations in liver or kidney enzyme levels or histopathological changes were observed, indicating a lack of hepatic or renal toxicity. This finding suggests that other components in the GSTH extract may counteract palmitic acid‐induced toxicity, or the concentration of palmitic acid present is efficiently metabolized and excreted, thus preventing its accumulation to harmful levels. In addition, other major constituents identified in the extract, such as linolenic acid and linoleic acid, are commonly found in natural sources and widely present in the human diet [48, 49]. These compounds are generally recognized as safe and are not associated with significant toxicity at normal consumption levels [48, 49].

Future studies should include pharmacokinetic and pharmacodynamic evaluations to further validate the safety and therapeutic potential of the GSTH extract. Pharmacokinetic studies would provide critical insights into the absorption, distribution, metabolism, and excretion (ADME) of the bioactive compounds present in the extract, helping to determine their bioavailability, half‐life, and potential for accumulation in target tissues. In addition, pharmacodynamic evaluations are necessary to understand the mechanism of action, dose–response relationship, and therapeutic window of the extract. Thus, further investigation would not only support a more accurate assessment of efficacy and toxicity but also guide appropriate dosage regimens for future preclinical and clinical investigations.

## 5. Conclusions

The findings of this study demonstrated that the Thai herbal recipe GSTH possessed significant antimalarial activity, as evidenced by its dose‐dependent suppression of parasitemia in a mouse model. The ethanolic GSTH extract achieved a suppression rate of 61.49% at the highest tested dose of 600 mg/kg body weight, indicating its potential as a natural antimalarial agent. Additionally, the acute toxicity assessment confirmed the safety of GSTH, with no observable adverse effects on physical behavior, body weight, liver and kidney enzyme levels, or histopathological parameters at a dose of 2 g/kg body weight. Overall, these results suggest that GSTH exhibits moderate in vivo antimalarial activity with acceptable safety in the tested model. However, additional preclinical studies, including mechanism of action investigations and chronic toxicity assessments, are necessary to further establish its pharmacological profile. Moreover, clinical trials are required to validate its efficacy and safety in humans.

## Author Contributions

Arisara Phuwajaroanpong: conceptualization, methodology, investigation, formal analysis, and writing–original draft. Chuchard Punsawad: conceptualization, methodology, investigation, formal analysis, writing–original draft, funding acquisition, and project administration. Walaiporn Plirat: conceptualization, methodology, investigation, formal analysis, and writing–original draft. Atthaphon Konyanee: conceptualization, methodology, investigation, formal analysis, and writing–original draft. Parnpen Viriyavejakul: investigation, formal analysis, and writing–review and editing. Abdi Wira Septama: formal analysis, and writing–review and editing. Prapaporn Chaniad: conceptualization, methodology, investigation, formal analysis, funding acquisition, project administration, writing–original draft, and writing–review and editing.

## Funding

This research was supported by the Walailak University Plant Genetic Conservation Project under the Royal Initiation of Her Royal Highness Princess Maha Chakri Sirindhorn (RSPG) (contract no. RSPG‐WU‐11/2567).

## Ethics Statement

The animal study protocol was approved by the Institutional Review Board (or Ethics Committee) of Walailak University, National Research Council of Thailand (NRCT) (protocol number: WU‐ACUC‐67006).

## Conflicts of Interest

The authors declare no conflicts of interest.

## Data Availability

The data associated with this study are included in this published article. Additional files are available from the corresponding authors upon request.
